# Evaluation of hypokalemia and potassium supplementation during administration of liposomal-amphotericin B

**DOI:** 10.3892/etm.2014.1534

**Published:** 2014-02-11

**Authors:** EISEKI USAMI, MICHIO KIMURA, TETSUFUMI KANEMATSU, SHINYA YOSHIDA, TAKAYUKI MORI, KEIJI NAKASHIMA, TOMOKO MATSUOKA, TOMOAKI YOSHIMURA, HIROMI MORI, TADASHI SUGIYAMA, HITOMI TERAMACHI

**Affiliations:** 1 Department of Pharmacy, Ogaki Municipal Hospital, Ogaki-shi, Gifu 503-8502, Japan; 2 Department of Pharmacy, Gifu Social Insurance Hospital, Kani-shi, Gifu 509-0206, Japan; 3 Laboratories of Clinical Pharmacy Practice and Social Science, Gifu Pharmaceutical University, Gifu-shi, Gifu 501-1196, Japan; 4 Clinical Pharmacy, Gifu Pharmaceutical University, Gifu-shi, Gifu 501-1196, Japan

**Keywords:** liposomal-amphotericin B, potassium supplementation, hypokalemia, risk factor

## Abstract

Patients prescribed liposomal-amphotericin B (L-AMB) frequently require supplemental potassium to prevent hypokalemia. The aim of this retrospective study was to examine the appropriate potassium supplementation conditions to treat hypokalemia induced by L-AMB. The subjects were 100 hematological patients who received L-AMB for the first time between April 2012 and March 2013. A total of seven patients were excluded. Of the remaining 93 patients, 48 (51.6%) were assigned to the group receiving supplemental potassium (supplementation group), and 45 (48.4%) were assigned to the group without potassium supplementation (non-supplementation group). Hypokalemia greater than grade 3 was exhibited by 50 of the 93 (53.8%) patients. Multivariate analysis revealed that the minimum serum potassium levels during L-AMB administration (≤2.98 mEq/l) were an independent factor significantly contributing to the effectiveness of potassium supplementation [odds ratio (OR), 3.62; 95% confidence interval (CI), 1.44–9.59; P<0.01]. In addition, multivariate analysis revealed that the serum potassium levels (≥2.83 mEq/l) prior to the potassium supplementation were an independent factor significantly contributing to the development of proper potassium supplementation (OR, 14.21; 95% CI, 1.95–310.72; P=0.02), and no significant difference was observed in the dosage of the potassium supplementation administered to the patients who recovered from hypokalemia and those who did not. In conclusion, it is necessary to begin potassium supplementation prior to the reduction of the serum potassium levels to <2.83 mEq/l. Potassium supplementation at an early stage of L-AMB treatment is important to prevent severe electrolyte abnormalities.

## Introduction

Invasive fungal infection is a major cause of morbidity and mortality in immunocompromised patients. Amphotericin B (AMB) possesses broad-spectrum antifungal activity and well-documented efficacy against Candida, Aspergillus and Cryptococcus infections (1). Liposomal-AMB (L-AMB), which was developed as a drug delivery system for AMB to reduce its adverse events (e.g., nephrotoxicity), replaced AMB (2) and is commonly used in clinical practice worldwide. L-AMB is recommended as a first-line drug for hematological patients in the guidelines of the Infectious Diseases Society of America (3,4). However, treatment with L-AMB may necessitate potassium supplementation to prevent hypokalemia. A previous study (5) reported the risk factors contributing to the occurrence of hypokalemia following L-AMB administration. The study revealed that patient’s serum albumin levels (≥2.82 mg/dl) at the start of L-AMB administration and history of hypokalemia prior to L-AMB administration were independent risk factors significantly contributing to the occurrence of hypokalemia (5). However, proper potassium supplementation for hypokalemia had not been sufficiently investigated. The present study therefore retrospectively examined proper potassium supplementation for hypokalemia induced by L-AMB

## Subjects and methods

### Subjects

**Figure 1:**
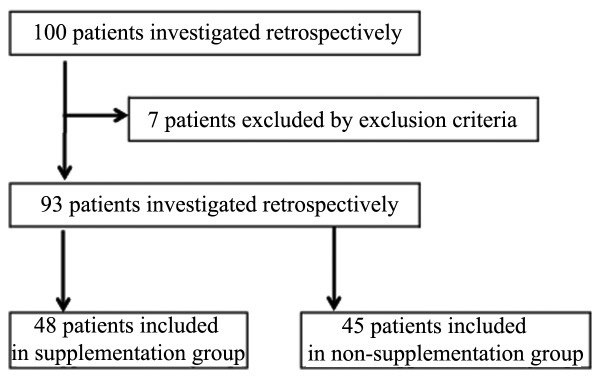
Subject selection and the number of subjects analyzed.

The subjects were 100 hematological patients who received L-AMB for the first time at Ogaki Municipal Hospital (Ogaki-shi, Japan) between April 2012 and March 2013. Seven patients were excluded due to prior or ongoing treatments that affect potassium levels (e.g., furosemide or fluid replacement during L-AMB administration), or as they had serum potassium levels <3.0 mEq/l (hypokalemia higher than grade 3) prior to the L-AMB administration. Of the remaining 93 patients, 48 (51.6%) were assigned to the group receiving supplemental potassium (supplementation group), and 45 (48.4%) were assigned to the group without potassium supplementation (non-supplementation group) (Fig. 1). L-AMB was administered once a day for 1–2 h. This study was reviewed and approved by the Ethics Committee at Ogaki Municipal Hospital.

### Background of the subjects

The backgrounds of the subjects treated with L-AMB were investigated for their gender, age, serum creatinine levels, treatment L-AMB dose, performance status (ECOG), underlying disease and prior use of antifungal drugs for a primary infection episode. The backgrounds between the supplementation or non-supplementation groups were compared.

### Incidence of hypokalemia and potassium supplementation

Incidences of hypokalemia greater than grade 3 (serum potassium levels <3.0 mEq/l) were identified and compared between the groups. Treatment dosing for potassium supplementation in the supplementation group was investigated.

### Change in the serum potassium levels

The change and the minimum levels of serum potassium during L-AMB administration were identified and compared between the groups.

### Investigation of the factors affecting the potassium supplementation during L-AMB administration

The factors affecting the potassium supplementation during L-AMB administration were examined between the groups.

### Investigation of the factors affecting proper potassium supplementation in the supplementation group

The serum potassium levels following the potassium supplementation were investigated, and the factors affecting proper potassium supplementation were examined. The proper potassium supplementation was defined as serum potassium levels that were maintained at >3.0 mEq/l (higher than grade 2) following the potassium supplementation.

### Method of data collection

Laboratory values in the form of biochemistry results were retrospectively identified from electronic medical charts. Data (including the age, serum creatinine levels, and L-AMB or potassium supplementation doses of the patients) were presented as the mean ± standard deviation. The grade of the hypokalemia was assessed in accordance with the Japan Clinical Oncology Group/Japan Society of Clinical Oncology Japanese version of the Common Terminology Criteria for Adverse Events, v4.0.

### Statistical analysis

Analyses were performed using JMP software (version 5.0.1J; SAS Institute Japan Ltd., Tokyo, Japan). Wilcoxon signed-rank test was used for comparison of the serum potassium levels prior to and following L-AMB administration. The Mann-Whitney U test was used for comparison of the backgrounds of the subjects between the groups. The recorded P-values were two-sided and values of <0.05 were considered to indicate a statistically significant difference. The areas under the receiver-operator characteristic (ROC) curves were calculated to estimate the accuracy and cut-off values for the continuous variables obtained by univariate logistic regression analysis. Subsequently, the data were analyzed using multivariate logistic regression analysis.

## Results

### Background of subjects

Table I summarizes the backgrounds of the subjects. The total L-AMB dosages for the supplementation and non-supplementation groups were 2485.1±1730.6 and 1485.6±1345.6 mg, respectively, and the treatment L-AMB doses were 125±30 and 110±25 mg/day, respectively. In addition, the duration of the L-AMB treatment was 19.8±14.2 and 12.9±8.4 days, respectively. The L-AMB was administered as a second-line therapy (following micafungin or caspofungin and others) in 61.0% (57/93) of the subjects and as a first-line therapy in 39.0% (36/93) of the subjects.

### Incidence of hypokalemia and potassium supplementation

The incidence of patients with hypokalemia greater than grade 3 (serum potassium levels <3.0 mEq/l) was 53.8% (50/93 subjects). Potassium supplementation was used to treat 51.6% of the subjects (48/93). The total potassium supplementation dosage was 519.6±506.1 mEq, and the treatment potassium supplementation dosage was 32.6±13.4 mEq/day. The duration of the potassium supplementation treatment was 14.4±14.0 days.

### Change in the serum potassium levels

**Figure 2:**
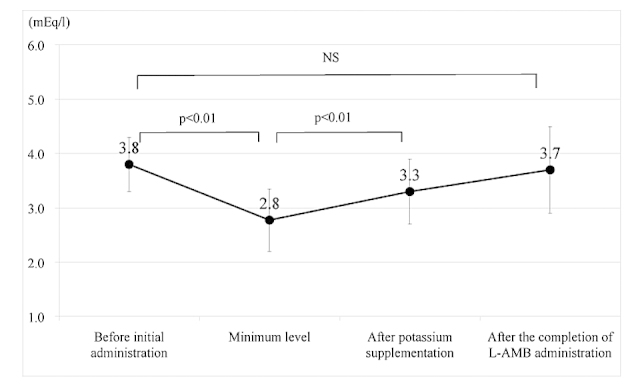
Change in the serum potassium levels during the L-AMB administration in the supplementation group. L-AMB, liposomal-amphotericin B; NS, not significant. Data are presented as the mean ± SD (n=48).

**Figure 3:**
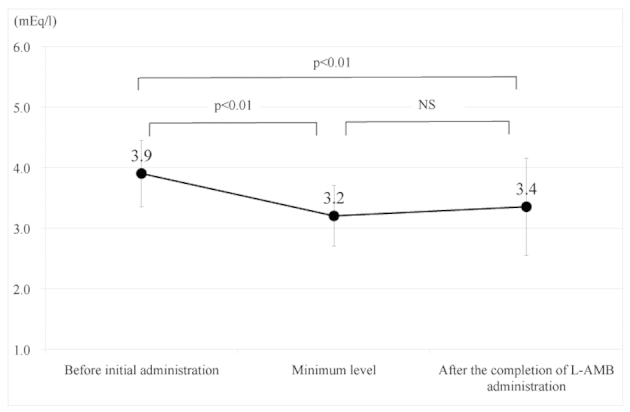
Change in the serum potassium levels during the L-AMB administration in the non-supplementation group. L-AMB, liposomal-amphotericin B; NS, not significant. Data are presented as the mean ± SD (n=45)

The change in the serum potassium levels during the L-AMB administration is shown in Figs. 2 and 3, divided into the two groups. In the supplementation group, the serum potassium levels significantly decreased from 3.8±0.5 to 2.8±0.6 mEq/l (P<0.01), when comparing the levels prior to the initial L-AMB administration with the minimum levels (at the time of the initialization of potassium supplementation). Subsequently, the levels significantly increased to 3.3±0.6 mEq/l following the potassium supplementation (P<0.01). In addition, the levels recovered to 3.7±0.8 mEq/l following completion of the L-AMB administration (2.9±2.6 days after completion). In the non-supplementation group, the serum potassium levels markedly decreased from 3.9±0.5 to 3.2±0.5 mEq/l (P<0.01), when comparing the levels prior to the initial L-AMB administration with the minimum levels. In addition, the levels only recovered to 3.4±0.8 mEq/l following completion of the L-AMB administration (2.0±1.5 days after completion) and a significant difference was identified compared with the levels prior to the L-AMB administration (P<0.01).

### Investigation of the factors affecting potassium supplementation during L-AMB administration

Nine factors affecting the differences between the groups were analyzed using univariate logistic regression analysis. The independent variables of the dosage data were analyzed as a continuous variable, and the results are shown in Table II. The total L-AMB dosage [odds ratio (OR), 67.97; 95% confidence interval (CI), 4.34–<1000; P<0.01], treatment L-AMB dose (OR, 25.57; 95% CI, 2.31–395.36; P=0.01), duration of the L-AMB treatment (OR, 224.62; 95% CI, 5.26–<1000; P<0.01), and minimum serum potassium levels during the L-AMB administration (OR 0.07; 95% CI 0.01–0.55; P=0.01) showed significant differences between the two groups. The areas under the ROC curves of these factors were 0.75, 0.63, 0.71 and 0.66, and the cut-off values were 2001.4 mg, 118.5 mg/day, 16.4 days and 2.98 mEq/l, respectively. Table III shows the results of the multivariate analysis based on the factors with P<0.25 by univariate logistic regression analysis. It revealed that the minimum serum potassium levels during L-AMB administration (≤2.98 mEq/l) were an independent factor significantly contributing to the effectiveness of potassium supplementation (OR, 3.62; 95% CI, 1.44–9.59; P<0.01).

### Investigation of the factors affecting proper potassium supplementation in the supplementation group

Twelve factors affecting the proper potassium supplementation were analyzed using univariate logistic regression analysis. The independent variables of dosage data were analyzed as a continuous variable, and the results are shown in Table IV. The serum potassium levels prior to the potassium supplementation showed a significant difference in the effectiveness of proper potassium supplementation (OR, 151.51; 95% CI, 12.60–733.52; P<0.01). The area under the ROC curve of this factor was 0.81 and the cut-off value was 2.83 mEq/l. Table V shows the results of the multivariate analysis based on the factors with P<0.25 by univariate logistic regression analysis. The serum potassium levels prior to the potassium supplementation (≥2.83 mEq/l) again showed a significant difference (OR, 14.21; 95% CI, 1.95–310.72; P=0.02). However, the duration of the potassium supplementation and the treatment L-AMB dose showed no significant difference in the effectiveness of the proper potassium supplementation.

## Discussion

Immunocompromised hematological patients frequently develop febrile neutropenia, thus empirical treatment with antifungal drugs is initiated prior to confirmation of a definitive diagnosis of a fungal infection (1). L-AMB possesses broad-spectrum antifungal activity and is a first-line indication against unconfirmed fungal infections in empirical therapy (2). Competitive studies with other antifungal drugs have demonstrated the efficacy of L-AMB (6,7).

The present study investigated hematological patients who were receiving L-AMB for the first time. Prior antifungal drugs used for the primary infection episode in the patients of the present study (61% of them used L-AMB in the change from other antifungal drugs) included micafungin and caspofungin. In addition, L-AMB was used in combination with intensive antibiotics, including carbapenem (82%) or glycopeptides (50%; data not shown). Infectious diseases in hematological patients may lead to a fatal outcome. Thus, infection control with antibiotics and antifungal drugs is critical.

L-AMB may reduce the levels of blood potassium by damaging the renal tubules (8,9). The incidence of hypokalemia in patients treated with L-AMB was reported as 36% by Ringden in 1994 (10) and as 51.3% by Sunakawa in 2012 (11). In the present study, hypokalemia greater than grade 3 occurred in 53.8% of the patients, making it an adverse event that requires attention. By comparing the serum potassium levels in patients of the two groups, the minimum levels in the supplementation group were significantly lower than those of the non-supplementation group, although no difference in them prior to the L-AMB administration was observed (mean±SD, 3.8±0.5 vs. 3.9±0.5 mEq/l; P=0.51). However, the serum potassium levels following the L-AMB administration were higher in the supplementation group than those in the non-supplementation group (3.7±0.8 vs. 3.4±0.8 mEq/l; P=0.04). Recovery of the patients from hypokalemia was more rapid in those in the supplementation group. Altogether, 51.6% of the subjects were treated with potassium supplementation. The supplementation group had a longer duration of L-AMB treatment and a greater total L-AMB dosage compared with those of the non-supplementation group. Following investigation of the factors affecting potassium supplementation during L-AMB administration, it was revealed that the minimum serum potassium levels during L-AMB administration (≤2.98 mEq/l) were an independent factor significantly contributing to the effectiveness of treatment with supplemental potassium. Investigation of the factors affecting the effectiveness of proper potassium supplementation in the supplementation group revealed that serum potassium levels prior to the potassium supplementation showed a significant difference between the patients that were successfully treated for hypokalemia and those that did not receive this treatment. This means that it is necessary to initiate potassium supplementation prior to reduction of the serum potassium levels to <2.83 mEq/l. Potassium supplementation from an early stage is important to maintain serum potassium levels at >3.0 mEq/l (higher than grade 2), thereby preventing proper potassium supplementation.

In conclusion, a periodic serum potassium levels monitor from the beginning of L-AMB administration and potassium supplementation from an early stage are important to prevent severe electrolyte abnormalities. Invasive opportunistic fungal infections are a serious cause of morbidity and mortality for immunocompromised hematological patients. Therefore, adverse events management of L-AMB is essential.

## Figures and Tables

**Table I:** Patient demographics and baseline characteristics.

	Potassium supplementation		
		
Demographics and characteristics	With	Without	P-value
Gender			
Male	29	25	0.63
Female	19	20	
Age (years; median)	68.3±12.3	67.2±14.9	0.61
Serum creatinine levels (mg/dl)			
Prior to L-AMB administration	0.70±0.62	0.76±0.63	0.65
Following L-AMB administration	0.91±0.71	0.86±0.81	0.75
L-AMB			
Total dosage (mg)	2485.1±1730.6	1485.6±1345.6	0.01
Treatment dose (mg/day)	125±30	110±25	0.01
Duration of treatment (days)	19.8±14.2	12.9±8.4	0.01
Potassium supplementation			
Total dosage (mEq)	519.6±506.1	-	
Treatment dose (mEq/day)	32.6±13.4	-	
Duration of treatment (days)	14.4±14.0	-	
Minimum serum potassium levels			<0.01
K ≥3.0 mEq/l	15	28	
K <3.0 mEq/l	33	17	
Performance status (ECOG)			0.82
0	12	14	
1	12	8	
2	9	8	
3	9	11	
4	6	4	
Underlying disease			
ML	12	19	
AML	22	10	
ALL	2	2	
MDS	3	3	
MM	4	8	
AA	3	3	
Others	2	0	
Prior antifungal drugs for the primary infection episode			
Micafungin	23	18	
Caspofungin	5	1	
Voriconazole	4	1	
Fluconazole	2	1	
Itraconazole	2	0	
Nothing	12	24	

Data are presented as n or the mean ± SD (n=93). L-AMB, liposomal-amphotericin B; K, serum potassium; ML, malignant lymphoma; AML, acute myeloid leukemia; ALL, acute lymphoblastic leukemia; MDS, myelodysplastic syndromes; MM, multiple myeloma; AA, aplastic anemia.

**Table II:** Univariate analysis of the factors affecting potassium supplementation during L-AMB administration (n=93).

Factor	OR	95% CI	P-value	AUC	Cut-off
Gender (female)	0.81	0.35–1.86	0.63		
Age	1.64	0.25–11.18	0.60		
Serum creatinine levels prior to L-AMB administration (mg/dl)	0.51	0.01–9.84	0.65		
Total L-AMB dosage (mg)	67.97	4.34–<1000	<0.01	0.75	2001.4
Treatment L-AMB dose (mg/day)	25.57	2.31–395.36	0.01	0.63	118.5
Duration of L-AMB treatment (days)	224.62	5.26–<1000	<0.01	0.71	16.40
Serum potassium levels prior to L-AMB administration (mEq/l)	0.51	0.06–3.78	0.51		
Minimum serum potassium levels during L-AMB administration (mEq/l)	0.07	0.01–0.55	0.01	0.66	2.98
PS ≥2	1.15	0.34–3.83	0.81		

L-AMB, liposomal-amphotericin B; OR, odds ratio; CI, confidence interval; AUC, area under the curve; PS, performance status.

**Table III:** Multivariate analysis of the factors affecting potassium supplementation during L-AMB administration (n=93).

Factor	OR	95% CI	P-value
Total L-AMB dosage (≥2001.4 mg)	3.23	0.67–17.47	0.14
Treatment dose (≥118.5 mg/day)	2.01	0.76–5.88	0.14
Duration of treatment (≥16.4 days)	1.39	0.29–6.17	0.66
Minimum serum potassium levels during L-AMB administration (≤2.98 mEq/l)	3.62	1.44–9.59	<0.01

L-AMB, liposomal-amphotericin B; OR, odds ratio; CI, confidence interval.

**Table IV:** Univariate analysis of the factors affecting proper potassium supplementation (n=48).

Factor	OR	95% CI	P-value	AUC	Cut-off
Gender (female)	1.43	0.38–6.16	0.61		
Age	0.44	0.11–10.25	0.62		
Serum creatinine levels prior to L-AMB administration (mg/dl)	6.07	0.04–>1000	0.61		
Total L-AMB dosage (mg)	0.15	0.01–2.72	0.19		
Treatment dose (mg/day)	0.14	0.01–1.48	0.11		
Duration of treatment (days)	0.23	0.01–8.71	0.39		
Potassium supplementation dose (mEq/day)	2.03	0.11–52.03	0.65		
Day of potassium supplementation start (days)	0.27	0.01–7.01	0.41		
Duration of potassium supplementation (days)	0.11	0.01–4.49	0.24		
Serum potassium levels prior to L-AMB administration (mEq/l)	2.60	0.21–37.67	0.46		
Serum potassium levels prior to potassium supplementation (mEq/l)	151.51	12.6–733.52	<0.01	0.81	2.83
PS ≥2	4.19	1.05–21.41	0.06		

L-AMB, liposomal-amphotericin B; OR, odds ratio; CI, confidence interval; AUC, area under the curve; PS, performance status.

**Table V:** Multivariate analysis of the factors affecting proper potassium supplementation (n=48).

Factor	OR	95% CI	P-value
Total L-AMB dosage (mg)	182.00	0.01–262.65	0.91
Treatment dose (mg/day)	0.74	0.12–4.08	0.73
Duration of potassium supplementation (days)	0.14	0.01–80.64	0.57
Serum potassium levels prior to potassium supplementation (≥2.83 mEq/l)	14.21	1.95–310.72	0.02
PS ≥2	3.04	0.62–18.23	0.18

L-AMB, liposomal-amphotericin B; OR, odds ratio; CI, confidence interval; PS, performance status.
